# Darbepoetin Alfa in Young Infants With Renal Failure: Single Center Experience, a Case Series and Review of the Literature

**DOI:** 10.3389/fped.2018.00398

**Published:** 2018-12-18

**Authors:** Anna Maria Libudzic-Nowak, Francois Cachat, Manuel Pascual, Hassib Chehade

**Affiliations:** ^1^Pediatric Nephrology Unit, Lausanne University Hospital (CHUV), Lausanne, Switzerland; ^2^Transplantation Center, Lausanne University Hospital (CHUV), Lausanne, Switzerland

**Keywords:** anemia, darbepoetin alfa, chronic kidney disease, infant, pediatric

## Abstract

**Background:** Anemia treatment in infants with advanced or chronic kidney disease (CKD) represents an important challenge to nephrologists. The use of darbepoetin alfa, a novel erythropoiesis stimulating agent, has largely replaced recombinant human erythropoietin in older children and in adults with CKD. However, studies reporting the use of darbepoetin alfa in infants below 1 year of age are rare.

**Case presentation:** We report the data of three infants with advanced stage kidney failure, aged 1, 4, and 7 months, who were treated with darbepoetin alfa and followed for 18–41 months. Hemoglobin levels increased in all three patients, reaching the target levels of 10.7–12 g/dl by 11, 19, and 22 weeks respectively, without any documented adverse effects. Patients younger than 1 year of age required a larger darbepoetin alfa dosage (ranged from 1.2 to 2.9 μg/kg per month) as compared to older children. A review of the literature found only three studies using darbepoetin alfa successfully in such young infants, with similar dosage and clinical success.

**Conclusion:** In these three patients with advanced kidney disease, darbepoetin alfa was effective in correcting anemia with no observed side effects. It reinforces its potential use in very young patients with advanced CKD.

## Background

Anemia in children with chronic kidney disease (CKD) is recognized as an important comorbidity factor and is associated with growth retardation and cognitive dysfunction (1), as well as increased cardiovascular risk (2, 3), and accelerated CKD progression (4). Recent studies in the pediatric CKD population have indicated an increased risk of hospitalization and death in children with anemia, as compared to children with appropriate hemoglobin levels (5, 6).

The National Kidney Foundation Kidney Disease Outcomes Quality Initiative (NKF KDOQI) guidelines recommend, in dialysed and non-dialysed children with CKD, a target hemoglobin level between 11 and 12 g/dl (7). According to the Kidney Disease Improving General Outcome (KDIGO) guidelines, anemia in children between 1 and 2 years of age with CKD, is defined as hemoglobin levels below 10.7 g/dl in boys and 10.8 g/dl in girls (8). It has been demonstrated that the use of erythropoiesis stimulating agents (ESA) for anemia in children with CKD decreases morbidity and mortality, reduces the need for blood transfusions, and improves growth (9).

Darbepoetin alfa (Aranesp®) is a hyperglycosylated epoetin analog with a similar mechanism of action as recombinant human erythropoietin (rHuEpo) and methoxy-polyethylene glycol-epoetin beta (Mircera®). The administration of darbepoetin alfa can be less frequent (weekly to monthly) than that of rHuEpo, for which the recommended administration frequency is 2–3 times per week (10). This latter dosing schedule can be cumbersome in infants and may lead to difficulties in obtaining target hemoglobin levels.

During the past 10 years, darbepoetin alfa has largely replaced rHuEpo in older children and in adults with CKD. However, data regarding the safety and efficacy of darbepoetin alfa in infants <1 year of age with CKD are rare (11–13). We hereby describe the successful use of darbepoetin alfa in three infants under the age of 8 months with advanced CKD and we performed a short non-systematic literature review regarding the use of darbepoetin alfa in infants <1 year of age with CKD.

## Patients and Methods

### Case Study

The clinical course and laboratory data of three male infants with advanced chronic kidney disease, aged 1, 4, and 7 months at the beginning of therapy, were retrospectively reviewed. Patients were followed for a minimum of 18 months (patient 3) and a maximum of 41 months (patient 1).

The baseline hemoglobin, iron, and ferritin levels were recorded (Table 1). Hemoglobin levels were measured before each darbepoetin alfa injection. Darbepoetin was first administered intravenously then subcutaneously in all patients. Target hemoglobin levels were established at 10.7–12 g/dl. All three patients were on oral iron supplement therapy [5 mg/kg of iron (III)-hydroxide polymaltose]. Adjustments of darbepoetin alfa dosage of ~25% were made if hemoglobin measurement remained below the target levels. Potential adverse effects (hypertension, seizures, local pain or inflammation, thrombocytosis) and unexpected reactions were all recorded during the follow-up.

**Table 1:** Characteristics of patients at baseline and at the last control.

	**Baseline**					**Last control**				
**Patient**	**Age (months)**	**Hemoglobin (g/l)**	**Creatinine (μmol/l)**	**Iron (μmol/l)**	**Ferritin (μg/l)**	**Age (months)**	**Hemoglobin (g/dl)**	**Creatinine (μmol/l)**	**Iron (μmol/l)**	**Ferritin (μg/l)**
1	7	9.3	135	11.4	27	48	12.1	142	15.9	146
2	1	10.7	249	14.7	96	20	11.8	445	15.6	109
3	4	7.7	235	15.2	485	22	11.0	150	N/A	N/A

### Literature Review

We searched the PubMed and Cochrane Library from the date of their inception to September, 12, 2018, to identify articles on darbepoetin alfa use in children aged <1 year using the keywords “darbepoetin” AND (“infant” OR “newborn”). We retrieved 33 articles, limited to humans and published in the English language. After reading the full text, three studies were included in our review. From the literature and appropriately selected papers, we also compared the pharmacology and pharmacokinetics of darbepoetin alpha in very young children receiving the drug for indications other than CKD.

## Results

**Figure 1:**
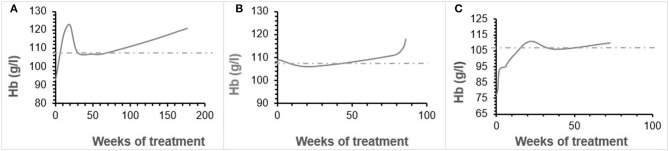
**(A–C)** Hemoglobin evolution in patient 1, 2, 3.

Patient 1 was a 7-month-old boy with CKD due to obstructive uropathy, with an estimated glomerular filtration rate (eGFR) of 30 ml/min × 1.73 m^2^. His anemia had been previously treated with epoetin beta (Recormon®) at the doses of 160 UI/kg twice a week. Despite epoetin alfa treatment associated with oral iron (4–6 mg/kg daily) and folic acid (1–2.5 mg/kg weekly) supplementations, adequate hemoglobin levels were not achieved. A switch to darbepoetin alfa at a mean dosage of 0.6 μg/kg twice per month allowed reaching hemoglobin target levels by week 11 (Figure 1A, Table 1). Treatment was interrupted for 2 months secondary to hemoglobin values above the target levels. It was then reintroduced at a slightly lower dose of 0.54 μg/kg twice per month, and then at a dose of 0.94 μg/kg monthly, with a favorable response (Tables 1, 2). After 41 months of follow-up, the hemoglobin level was 12 g/dl (Table 2). No adverse reaction was recorded during the treatment period.

**Table 2:** Darbepoetin alfa doses and intervals.

	**Before achieving Hb target**		**At the last control**	
**Patient**	**Dose of darbepoetin (μg/kg) Mean (*SD*) Range**	**Interval between doses (weeks) Mean (*SD*) Range**	**Dose of darbepoetin (μg/kg)**	**Interval between doses (weeks)**
1	0.6 (0.02) 0.58–0.62	2.2 (0.44) 2–3	0.94	4
2	1.02 (0.19) 0.75–1.2	2.83 (0.98) 2–4	1.0	2
3	1.1 (0.16) 1.0–1.45	2 (0) 2	1.0	2

Patient 2 was a 1-month-old newborn with severe renal failure due to obstructive uropathy (eGFR < 10 ml/min × 1.73 m^2^). He was temporarily dialysed for 2 weeks during the neonatal period because of fluid overload and severe hyponatremia. Treatment with darbepoetin alfa was initially started at a dose of 0.8 μg/kg subcutaneously twice per month, and then increased to a mean dose of 1 μg/kg, given at a mean interval of 2.8 weeks (Figure 1B, Table 2) together with oral iron supplementation (5 mg/kg/d). This allowed reaching his hemoglobin target level at week 19. After 19 months of follow-up the hemoglobin level was 11.8 g/dl. There were no adverse effect recorded during therapy.

Patient 3 was a 4-month-old infant with advanced renal failure (eGFR < 20 ml/min × 1.73 m^2^), secondary to obstructive uropathy. Darbepoetin alfa treatment was started at a dose of 1 μg/kg twice a month, associated with oral iron supplementation (5 mg/kg/d). Target hemoglobin level was reached at week 22, with a mean dose of darbepoetin alfa of 1.1 μg/kg every other week (Figure 1C, Table 2). After 18 months of follow-up, the hemoglobin level was stable at 11 g/dl (Table 1). No side-effects were recorded during follow-up. The iron stores evaluated at the beginning of the treatment were low for all of infants. Oral iron substitution allowed correction of iron deficit in all patients.

## Discussion

We report the cases of three infants aged 1–7 months with anemia and advanced CKD who were successfully treated with darbepoetin alfa. Although darbepoetin alfa has been effectively used in children with CKD above the age of 1 year (14–16), studies reporting the use of this treatment in a younger population remain sparse. Durkan et al. (12) were the first to demonstrate the efficacy of darbepoetin alfa in a study including six infants with anemia due to CKD, aged 0.9–7.9 months with a mean weight of 4 kg and creatinine levels between 126 and 340 μmol/l. This retrospective study revealed an optimal response to treatment with darbepoetin alfa at a relatively small dose (0.25 μg/kg/week) in three patients, but surprisingly no effect on hemoglobin level in the three other infants with medical complications, and this despite adjusting dosage up to 1.1 μg/kg/week. All infants were treated for a 20-week period at 1 to 4 week intervals between doses. In this study the hemoglobin target was fixed at 10–11 g/dl based on NKF K/DOQI guidelines (2000) and the recommendations of Royal College of Physicians and the London Renal Association, which is lower than the hemoglobin target in our study. The authors concluded that darbepoetin alfa can be successfully administered in infants, but the dosage needs to be tailored to each individual with varying response, potentially depending on general medical condition.

Rijk et al. (11) reported, in a retrospective multicenter study, 19 children on nightly intermittent peritoneal dialysis, with a mean age of 6.8 years (range 0–17 years). All children received darbepoetin alfa intraperitoneally for a median period of 13 months. The initially administered dose of darbepoetin alfa was 0.45 μg/kg/week, adjusted monthly, and reaching a dosage of 0.63 μg/kg/week to allow keeping stable hemoglobin levels. The following adverse effects were reported: headache, hypertension, and peritonitis (15 episodes). The authors concluded that intraperitoneal treatment with darbepoetin alfa is an effective alternative to rHuEpo treatment, with less frequent administration in children with CKD. Unfortunately, the exact age for each individual was not described, and the exact number of patients <1 year of age and their precise clinical outcome are not available.

Recently, Schaefer et al. (13), in a multicenter phase IV prospective study, reported that darbepoetin alfa to be very effective in treating anemia. The study included 13 infants <1 year of age with CKD. Twelve out of 13 patients were on darbepoetin treatment at the inclusion. After enrolment, they received darbepoetin alfa at a mean dosage of 1.7–3.2 μg/kg/month subcutaneously or intravenously, which was higher than doses administrated to older children (1.2–2.7 μg/kg/month for those aged 1–17 years). Darbepoetin alfa dosage was also higher if administrated intravenously or in dialysed patients. A significant number of adverse events (10 cases) were reported in this age subgroup (hypertension, diarrhea and gastroenteritis, convulsion, pyrexia, catheter-site infection, device-related infection, and peritonitis). The mean hemoglobin levels rose between 3 and 6 months after the beginning of the treatment and remained stable during the follow-up of maximum 2 years (after 15 months of follow-up data were available for 6 patients younger than 1 year at the enrolment). These results confirm the efficacy of darbepoetin alfa in correcting anemia in infants <1 year of age with CKD. Table 3 summarizes the 3 above-mentioned studies.

**Table 3:** Summary of studies reporting the use of darbepoetin alfa in infants <1 year of age.

**Authors/Year of the study**	**Type of the study**	**No of patients <1 year of age**	**Treatment indication**	**Doses (μg/kg/month)**	**Administration frequency**	**Treatment duration/ Follow-up**	**Target Hb (g/dl)**	**Adverse effects (number of patients)**
Durkan et al. (12)	Retrospective case-series	6	CKD-induced anemia	1.0–4.8	1–4 weeks	20 weeks	10–11	Injection site pain (6)
Rijk et al. (11)	Retrospective two-center single-arm	Not defined (19 patients of age range 0–17 years)	CKD-induced anemia	1.8–6.88	1–2 weeks	Range 2–40 months (median 13.4 months)	10.9–12.8	Hypertension (3) Headache (1) Peritonitis (15)
Schaefer et al. (13)	Prospective multicenter observational	13	CKD- induced anemia	1.7–3.2	2x/week–q 2 weeks	Range 1–117 weeks (mean 88 weeks)	10–12	Peritonitis (1)Pyrexia (1) Hypertension (1) Gastroenteritis (3) Convulsions (1) Catheter site infection (2) Device-related infection (1)

Darbepoetin alfa has been used in young infants for indications other than CKD. In a randomized placebo-controlled study, Ohls et al. (17) showed that darbepoetin alfa was successful in minimizing transfusion needs and maintaining red cell mass in preterm infants <48 h of age with a birth weight of 500–1,250 g. Preterm infants treated with darbepoetin alfa also presented better neurocognitive outcomes at 18 to 22 months and at 3.5 to 4 years, compared to placebo treated neonates (18, 19). In that specific group, darbepoetin alpha was given at a starting dose of 10 μg/kg every week subcutaneously. A recent Cochrane review (20) evaluated effectiveness of DA in low birth weight newborns with the significative outcome on RBC transfusions and necrotising enterocolitis during neonatal period as well as neurodevelopment until the age of 2.

More recently, darbepoetin alfa has also been used as a neuroprotective agent in conjunction with cooling therapy for hypoxic-ischemic encephalopathy in neonates. Baserga et al. (21) found darbepoetin alpha to be safe in critically ill neonates undergoing hypothermic therapy, at doses varying between 2 and 10 μg/kg. Further studies are necessary to assess if darbepoetin contributes to improvement of neurological outcomes. Darbepoetin alfa has been used for many years in older pediatric cancer patients to treat chemotherapy-induced anemia, at a recommended dosage of 2.25 μg/kg weekly or 500 μg every 3 weeks subcutaneously (22–24). Dosage of darbepoetin alpha seems to be similar in children with anemia secondary to CKD or to prematurity, but experience is sparse and target hemoglobin are different from other indication, making comparisons difficult. In our patients we observed a satisfactory response, without any documented adverse effects during a follow-up of 18–41 months.

Interestingly and as recently shown by Schaefer et al. (13), we found that patients younger than 1 year of age required a larger darbepoetin alfa dosage than older ones. In our observation the range of darbepoetin alfa dosage varied from 1.2 to 2.9 μg/kg per month, close to the dosage reported in Schaefer's study (1.7–3.2 μg/kg per month for subgroup of infants aged <1 year). This is higher than the recently recommended weekly dosage of 0.41 μg/kg (25–75th percentiles: 0.25–0.82 μg/kg) in pediatric patients with CKD aged 1–18 years (14).

The most frequently reported adverse effects of darbepoetin alfa are injection site pain, fever, headache, flu-like symptoms, hypertension, and thrombosis of vascular access, but their frequency compared favorably to another ESA (10–14). In children, subcutaneous injection of darbepoetin alfa has been reported by Schmitt et al. (25) to be more painful than those of epoetin-beta in a prospective, randomized, double-blind trial in 13 pediatric patients. In our study, we collected retrospectively all data concerning potential adverse events. Patient 1 and 2 were treated for hypertension, which started before darbepoetin alpha injections and was not worsen by the latter treatment. No injection site pain occurred in our patients, mainly because all of them received the darbepoetin intravenously. We observed no fever, seizure, headaches. However, it should be recognized that the short follow-up time and the very small number of patients preclude any firm conclusion regarding adverse effects of darbepoeitin alpha in this very young population with advanced CKD.

In conclusion, we report our results corroborate and extend the recent findings of Schaefer et al. (13). Prospective studies with larger sample size and longer follow-up should be conducted, to ensure darbepoetin alfa long lasting efficacy and the absence of severe or new adverse effects in young infants.

## Availability of Data and Materials

The data sets used for this article are available from the corresponding author upon request.

## Consent to Publish

Parents of the three infants have given their consent to participate in this study and publish the results.

## Ethics Statement

According to the Swiss ethics guidelines, the use of experimental therapy, done for a pure therapeutic goal (without a research goal) is based on the best judgment of the clinician for the benefit of a given patient and is being decided on a case by case basis. Our article is a retrospective analysis of 3 cases of experimental therapy used for the benefit of the patient only, and without a primary research goal. In such a condition, the Local Ethics Committee intervention is not necessary.

## Author Contributions

AL-N collected and analyzed the data, wrote the manuscript as submitted and agrees to be accountable for all aspects of the work. FC and MP participated in collecting the data and critically reviewed the manuscript. HC collected and analyzed the data, wrote the manuscript and critically reviewed the manuscript. All authors approved the final version of the manuscript.

### Conflict of Interest Statement

The authors declare that the research was conducted in the absence of any commercial or financial relationships that could be construed as a potential conflict of interest.

## Abbreviations

CKD: chronic kidney disease
ESA: erythropoiesis stimulating agents
eGFR: estimated glomerular filtration rate
NKF KDOQI: National Kidney Foundation Kidney Disease Outcomes Quality Initiative
rHuEpo: recombinant human erythropoietin.
